# Multi-Locus Genome-Wide Association Study Identifies Stable Quantitative Trait Nucleotides and Candidate Genes for Seed Protein Content in an Early-Maturing Soybean Recombinant Inbred Line Population

**DOI:** 10.3390/plants15213298

**Published:** 2026-10-26

**Authors:** Quanzhong Dong, Fulin Shan, Bo Hu, Mingming Zhang, Hong Xue, Weiwei Li, Huan Song, Ming Li, Xue Qu, Wen-Xia Li, Hailong Ning

**Affiliations:** 1 Keshan Branch of Heilongjiang Academy of Agricultural Sciences, Keshan, Qiqihar 161600, China; 2 Key Laboratory of Soybean Biology, Ministry of Education, Harbin 150030, China; 3 Key Laboratory of Soybean Biology and Breeding/Genetics, Ministry of Agriculture and Rural Affairs, Harbin 150030, China; 4 College of Crop Science, Northeast Agricultural University, Harbin 150030, China

**Keywords:** soybean, seed protein content, multi-locus GWAS, candidate gene, haplotype variation

## Abstract

Soybean seed protein content is a polygenic quantitative trait, and dissecting its genetic architecture is critical for advancing high-protein soybean molecular breeding. In this study, an F_5_ recombinant inbred line (RIL) population with 174 lines derived from a cross between high-protein cultivar Kedou 48 and low-protein cultivar Kedou 61 was phenotyped for seed protein content across four distinct growing environments. Genotyping was performed using 25,663 high-quality single nucleotide polymorphisms (SNPs), followed by multi-locus genome-wide association studies (GWAS). Phenotypic data revealed seed protein content ranged from 31.24% to 46.65% across the population, with a broad-sense heritability of 0.89. In total, 54 quantitative trait nucleotides (QTNs) linked to seed protein content were detected via 11 multi-locus GWAS algorithms; among these, 16 QTNs were classified as stable QTNs because they were detected by at least two GWAS models within the same environment and/or in at least two environments, and 33 QTNs overlapped with previously published seed protein quantitative trait loci (QTLs). Integrating linkage disequilibrium (LD) decay estimation, seed tissue expression profiles, parental resequencing-derived non-synonymous variants, and germplasm haplotype characterization, three candidate genes were prioritized: *Glyma.08G246400* (AFP3/NINJA-like transcriptional repressor), *Glyma.09G154600* (LRR receptor-like protein kinase), and *Glyma.19G224000* (translation initiation factor IF-2). Haplotype analyses using eligible accessions from the collaborative 2898-accession soybean resequencing panel identified significant haplotype–protein associations for all three genes after adjustment for germplasm type, with divergent haplotype distributions among Soja, landraces, and improved cultivars. This work provides stable QTNs supported by repeated detection, three biologically plausible candidate genes, and protein-associated haplotypes for further mechanistic and breeding evaluation in high-protein early-maturing soybean.

## 1. Introduction

Soybean (*Glycine max* (L.) Merr.) is a globally vital grain and oil crop, with seeds containing approximately 40% protein, which serves as an irreplaceable raw material for food processing, animal feed, and plant protein production. Enhancing seed protein concentration can relieve the global shortage of dietary plant protein. Nevertheless, seed protein content is a complex polygenic trait that shows negative genetic correlations with oil content and grain yield, creating major bottlenecks for high-protein cultivar improvement [1]. Unraveling the genetic basis governing seed protein accumulation and mining stably inherited superior alleles are essential to break these trade-offs and implement molecular design breeding for high-protein soybean varieties.

High-density genotyping arrays and complete soybean reference genomes have made QTL linkage mapping and GWAS the primary tools to dissect complex seed quality traits [2]. As of July 2026, the SoyBase database archives 255 biparental linkage QTLs and 260 GWAS-derived QTNs associated with soybean seed protein [3]. Recent studies have identified 28 protein-related QTLs using biparental RIL populations [4,5,6], 18 QTNs from four-way RIL panels [7], and 121 QTNs from natural germplasm collections [8,9]. Despite abundant documented loci, most reported QTLs/QTNs lack consistent performance under varied genetic backgrounds and field environments, limiting their practical breeding application. Multi-environment phenotypic evaluation combined with multiple statistical models can separate genetic and environmental variance components and improve the detection accuracy of environmentally stable QTNs, offering more robust genomic resources for soybean protein improvement [10].

At the molecular level, several functionally characterized genes have clarified how soybean seed protein content is coupled with carbon allocation, oil accumulation, and seed development. *GmSWEET39* underlies a major protein–oil QTL and alters sugar transport from maternal tissues to the developing embryo, with contrasting alleles favoring protein or oil accumulation [11]. *POWR1*, a CCT-domain domestication gene, pleiotropically regulates seed protein, oil, seed weight, and yield [12]. *GmST05* influences seed size together with oil and protein contents, partly through regulation of *GmSWEET10a* [13], whereas *GmSop20* (the *qSOP20* locus) coordinates the seed oil-to-protein ratio through *GmSWEET10a*-mediated sugar allocation [14]. More recently, *SW14* was shown to positively regulate seed weight and protein content while negatively affecting oil content [15], and *PC08* was identified as a positive regulator of seed storage protein accumulation through enhanced ABA biosynthesis [16]. *FA9*, encoding a SEIPIN homolog, primarily regulates fatty acid accumulation but also exerts pleiotropic effects on seed protein content and seed size [17]. Together, these studies demonstrate that soybean seed protein accumulation is controlled by interconnected pathways involving assimilate transport, hormone signaling, lipid metabolism, and seed development.

Methodologically, multi-model GWAS has become increasingly important for improving the robustness of QTN detection for complex soybean traits. Zhang et al. (2018) applied five multi-locus methods—mrMLM, FASTmrMLM, FASTmrEMMA, pLARmEB, and ISIS EM-BLASSO—to seed protein content in a four-way RIL population evaluated across 20 environments and detected 129 QTNs, including 22 loci repeatedly identified by multiple methods or environments [18]. Zhang et al. (2020) subsequently integrated six multi-locus algorithms, including pKWmEB, into the mrMLM v4.0 platform, providing a unified framework for multi-locus association analysis [19]. FarmCPU, proposed by Liu et al. (2016), iteratively uses fixed- and random-effect models to reduce confounding and improve statistical power [20], while BLINK, developed by Huang et al. (2019), further improves computational efficiency and association power for large datasets [21]. Li et al. (2022) developed 3VmrMLM and its IIIVmrMLM software to dissect main-effect QTNs together with QTN-by-environment and epistatic effects [22], and Wang et al. (2025) introduced Fast3VmrMLM to accelerate genome-wide scanning and gene mining in large-scale datasets [23]. These complementary frameworks provide a strong rationale for cross-model validation when prioritizing stable loci.

Despite these advances, three limitations remain in soybean seed protein genetics: (1) relatively few loci have been repeatedly detected across multiple environments and complementary GWAS models; (2) integration of GWAS signals with expression, functional variation, and haplotype evidence remains limited, restricting biological interpretation; and (3) haplotype-frequency and geographic-distribution patterns across Soja, landraces, and improved cultivars remain insufficiently characterized. To address these gaps, this study constructed an F_5_ biparental RIL population from two cultivars with divergent seed protein levels, collected multi-location phenotypic data across four environments, and performed GWAS using 11 complementary models with high-density SNP markers. Through sequential filtering based on locus stability, LD boundaries, seed expression, parental coding variants, and haplotype–phenotype associations, we aimed to: (1) identify stable QTNs supported by repeated detection across complementary GWAS models and/or environments; (2) screen functionally relevant candidate genes with multi-omics supporting evidence; and (3) characterize haplotype-frequency differences among germplasm types and their geographic distributions to identify protein-associated haplotypes for further breeding evaluation.

## 2. Results

### 2.1. Phenotypic Variation and Genetic Parameter Estimation

**Figure 1:**
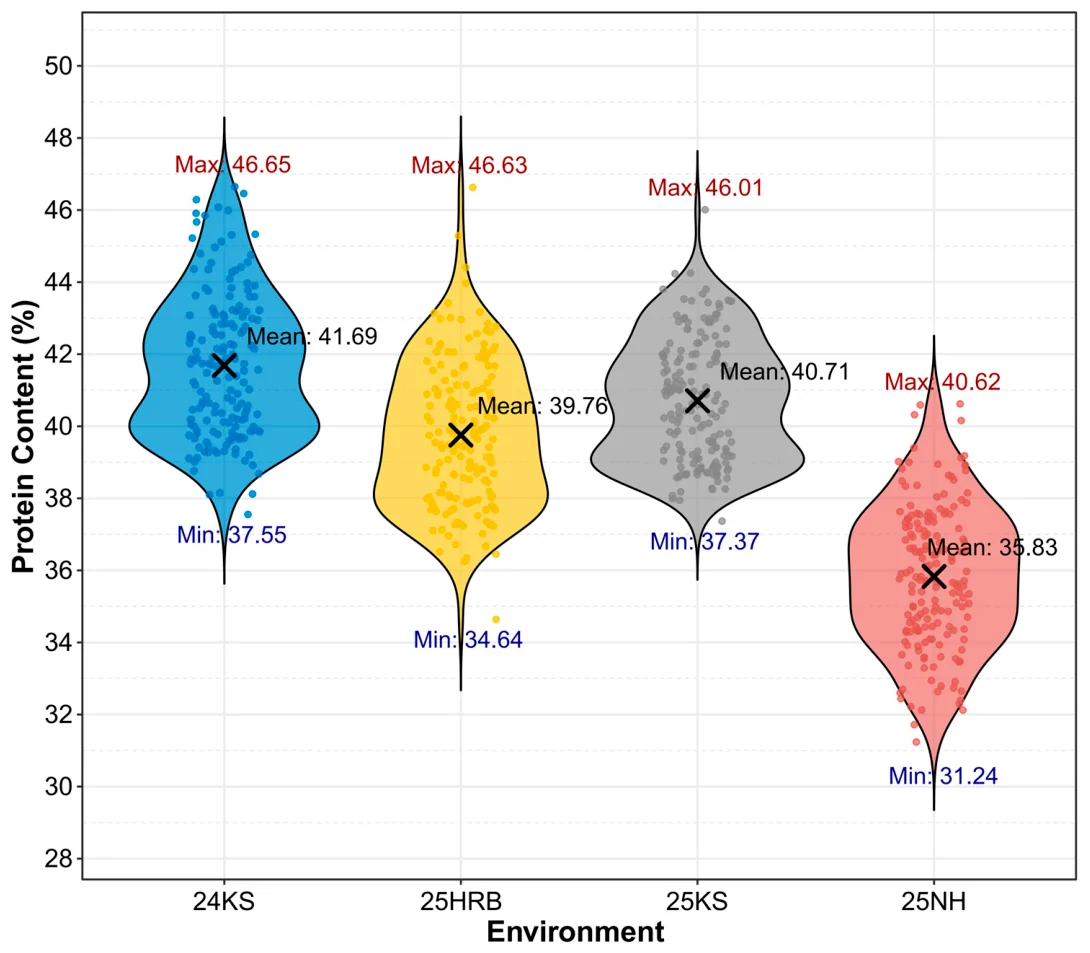
Violin plots showing seed protein content distribution of 174 RIL lines across four field environments. Black crosses represent population mean values; red and blue text denote the maximum and minimum values, respectively. Blue, yellow, gray, and red violins and points represent 24KS, 25HRB, 25KS, and 25NH, respectively.

Seed protein content measured across four environments exhibited a normal distribution, satisfying the statistical assumptions for GWAS (Figure 1). Descriptive statistics for each environment are summarized in Table S1. The ANOVA results are summarized in Table 1. The overall seed protein range of the RIL population was 31.24–46.65%, with a population-wide average of 39.49% and a total range of 15.41%, indicating abundant heritable variation. The 24KS environment produced the highest average protein content (41.69%), while 25NH showed the lowest mean value (35.83%). Coefficients of variation ranged from 4.15% to 5.49%, with skewness and kurtosis values close to standard normal distribution parameters.

### 2.2. Population Genetic Structure

**Figure 2:**
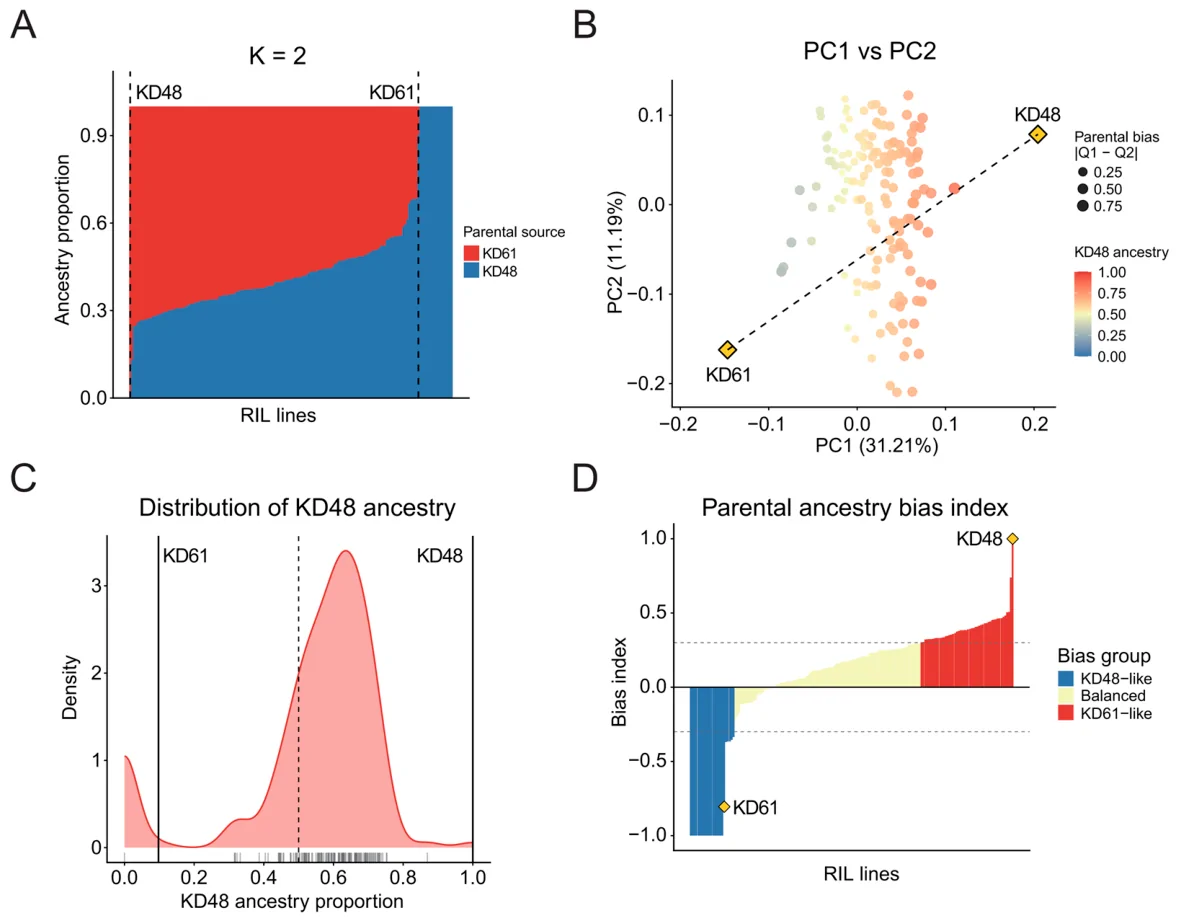
Population genetic structure analysis of the RIL panel. (**A**) Admixture ancestry plot at K = 2; each vertical bar represents one RIL line. (**B**) PCA scatter plot of PC1 vs. PC2, colored by KD48 ancestry proportion. (**C**) Density distribution of KD48 ancestry proportion. (**D**) Parental ancestry bias index for all lines. Dashed lines mark the parental reference positions in (**A**), connect the parental genotypes in (**B**), indicate a KD48 ancestry proportion of 0.5 in (**C**), and mark the ±0.3 bias-index thresholds in (**D**).

ADMIXTURE cross-validation (CV) error decreased most sharply from K = 1 to K = 2, followed by comparatively small changes across higher K values, with the absolute minimum observed at K = 10 (Figure S1). Given the biparental origin of the RIL population, additional clusters beyond K = 2 were interpreted as finer subdivisions of recombined parental ancestry rather than independent founder populations. Therefore, K = 2 was retained as the most parsimonious and biologically interpretable representation of the two parental genetic backgrounds (Figure 2A). All RIL lines displayed a continuous gradient of mixed ancestral components without discrete subpopulations. The density distribution of KD48 ancestry proportions showed a unimodal peak centered near the 0.5 balanced threshold (Figure 2C). Ancestry bias index values were continuously distributed across the population, allowing classification into KD48-like, balanced, and KD61-like subgroups (Figure 2D). PCA results indicated PC1 and PC2 explained 31.21% and 11.19% of total genetic variation, respectively; the first three PCs cumulatively captured 46.40% of population diversity. The two parental genotypes occupied opposite ends of the PCA scatter plot, with all RIL lines evenly distributed between them (Figure 2B). PC1–PC3 were incorporated as covariates in all GWAS models to eliminate stratification bias.

### 2.3. Multi-Locus GWAS Detects Stable Seed Protein QTNs

**Figure 3:**
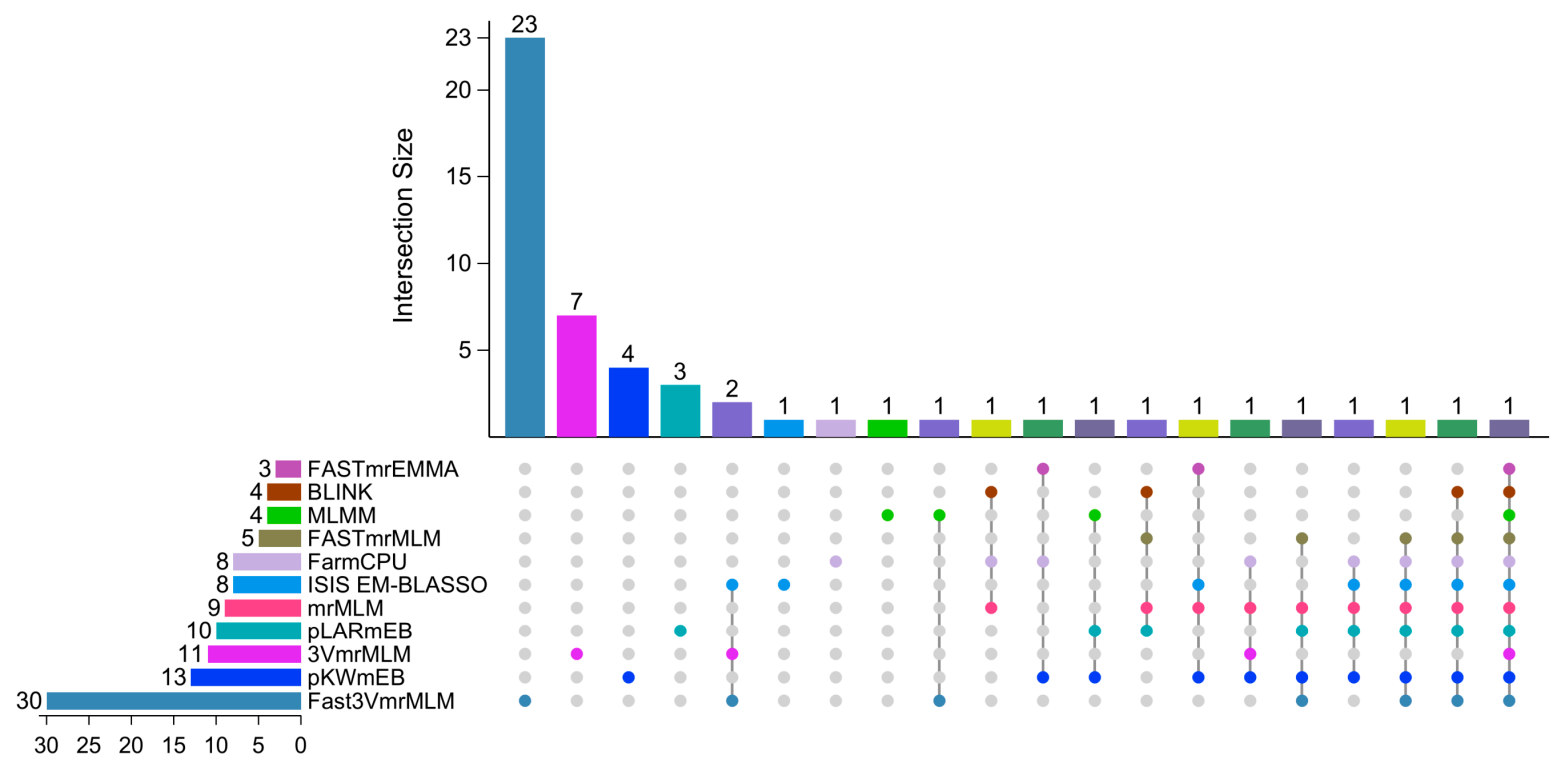
UpSet plot showing the numbers of unique QTNs detected by the 11 GWAS models and their intersections.

**Figure 4:**
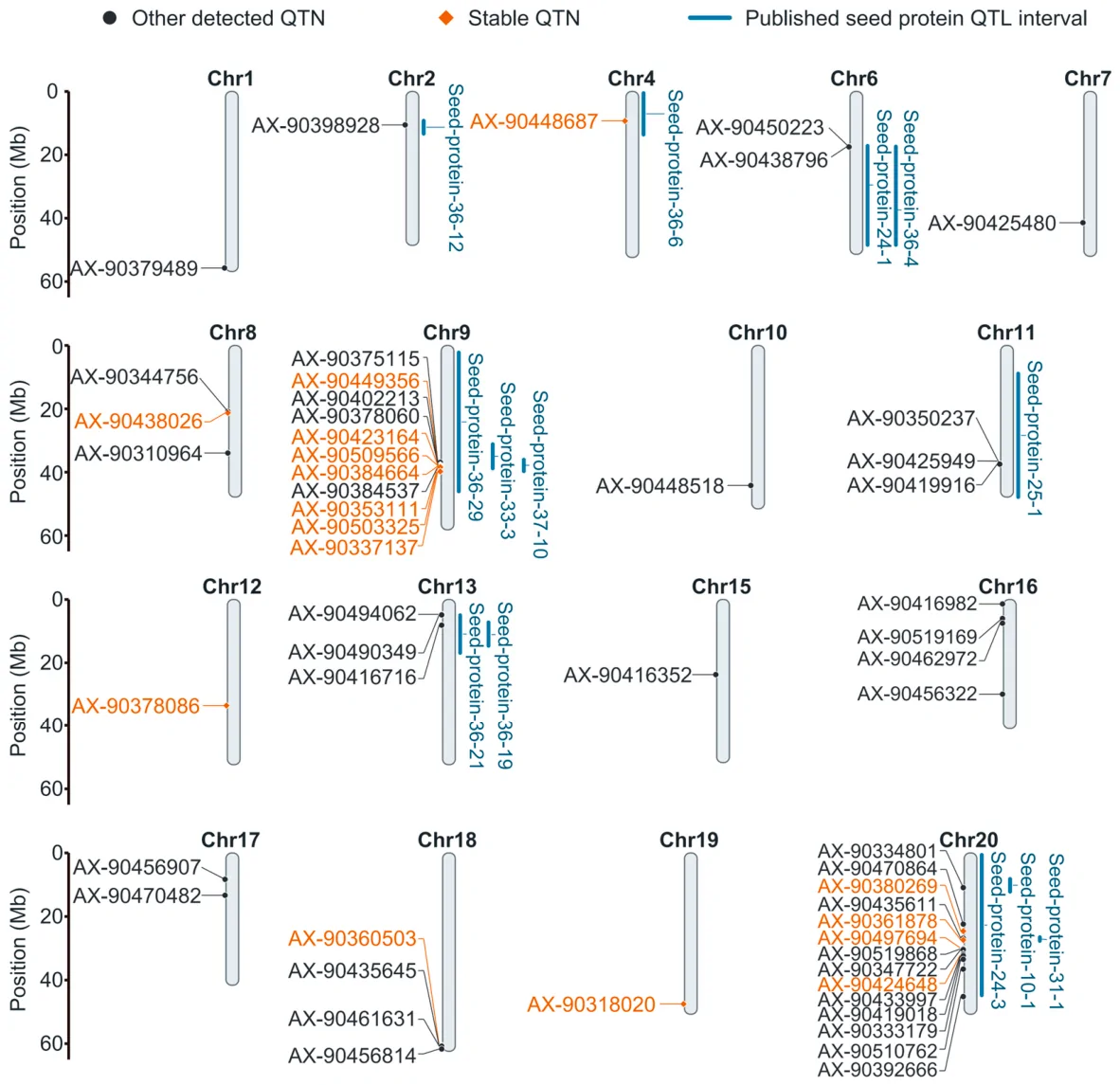
Chromosomal map illustrating physical positions of all 54 detected QTNs and overlapping published seed protein QTL intervals.

Across all 11 multi-locus models, a total of 54 unique QTNs associated with seed protein content were identified and distributed over 17 soybean chromosomes, with individual locus phenotypic variance explained (PVE) ranging from 0.39% to 61.26%. The six mrMLM-package methods detected 21 unique QTNs; the three GAPIT3 models (BLINK, FarmCPU, and MLMM) detected 12; and 3VmrMLM and Fast3VmrMLM detected 38. These sets overlapped across model groups (Figure 3; Table S2). Using the predefined repeat-detection criterion, 16 QTNs were classified as stable QTNs: 12 were detected by at least two GWAS models within a single environment, whereas four (AX-90438026, AX-90353111, AX-90380269, and AX-90497694) were detected in at least two environments. Of these four cross-environment QTNs, AX-90380269 and AX-90497694 were also supported by multiple GWAS models (Table S2). Comparative alignment with SoyBase QTL data revealed 33 QTNs overlapped with previously documented seed protein intervals. The 16 stable QTNs were located on chromosomes 4, 8, 9, 12, 18, 19, and 20, forming core genomic intervals for candidate gene mining (Figure 4). QQ plots comparing observed and expected *p*-values for BLINK, FarmCPU, and MLMM across the four field environments are presented in Figure S2A–C, respectively. The four blue QQ plots in Figure S2D–G correspond to the mrMLM algorithm for 25HRB, 25NH, 24KS, and 25KS, respectively.

### 2.4. Hierarchical Screening of Candidate Genes

**Figure 5:**
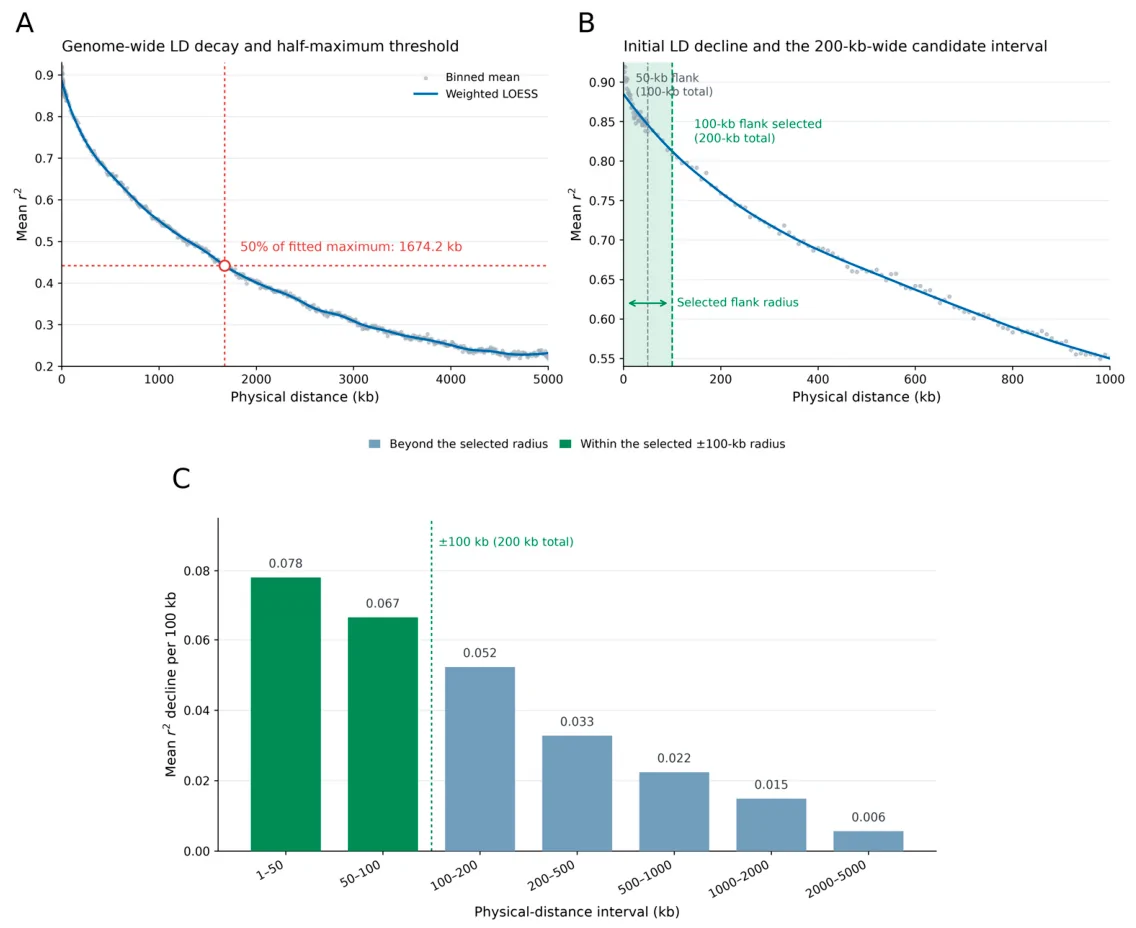
Genome-wide linkage disequilibrium (LD) decay and rationale for the stringent QTN-centered candidate-gene interval in the RIL population. (**A**) Genome-wide binned mean r^2^ values and the pair-count-weighted LOESS fit; red dashed lines indicate 50% of the fitted maximum (r^2^ = 0.442) and the corresponding distance (1674.2 kb). (**B**) Enlarged view of the initial decline. The green region represents the selected maximum QTN-to-gene distance of 100 kb (±100 kb; 200 kb in total), whereas the gray line indicates the narrower ±50-kb alternative. (**C**) Distance-normalized mean r^2^ decline across successive physical-distance intervals. The ±100-kb interval was used as an operational prioritization window and not as the genome-wide LD decay distance.

The sequential gene screening workflow is outlined in Table 2. The fitted genome-wide LD curve reached half of its maximum at 1674.2 kb (Figure 5A). Because applying this megabase-scale distance to each QTN would produce an excessively broad candidate-gene search, a stringent QTN-centered interval of ±100 kb (200 kb in total) was used for proximal-gene prioritization, consistent with soybean GWAS practice [24]. This interval retained the 50–100 kb segment, where fitted mean r^2^ remained high (0.846–0.813) and the distance-normalized decline (0.067 per 100 kb) remained close to that over 1–50 kb (0.078); a 100-kb total interval (±50 kb) would have excluded this segment (Figure 5B,C). Using this operational interval, 179 annotated genes were retrieved around the stable QTN peak SNPs (Table S3). Tissue expression profiling retained 40 genes with FPKM ≥ 5 in at least one of the five developing-seed stages (seed-1 to seed-5); the tissue expression heatmap is shown in Figure S3. Analysis of the independently generated KD48 and KD61 parental resequencing data further narrowed this set to seven unique genes carrying coding-region non-synonymous mutations (Table S4). Subsequent haplotype–phenotype testing adjusted for germplasm type prioritized three genes showing significant haplotype–protein associations: *Glyma.08G246400*, *Glyma.09G154600*, and *Glyma.19G224000* (Figure S8). GO annotations suggested distinct putative functions for the three candidates (Table S5): *Glyma.08G246400* was annotated as an AFP3/NINJA-like transcriptional repressor with GO terms related to ABA signaling and nitrogen metabolism; *Glyma.09G154600* was annotated as an LRR receptor-like protein kinase associated with protein phosphorylation and nutrient signaling; and *Glyma.19G224000* was annotated as translation initiation factor IF-2, with functions related to protein biosynthesis and ribosomal assembly.

### 2.5. Haplotype-Phenotype Associations for Three Candidate Genes

**Figure 6:**
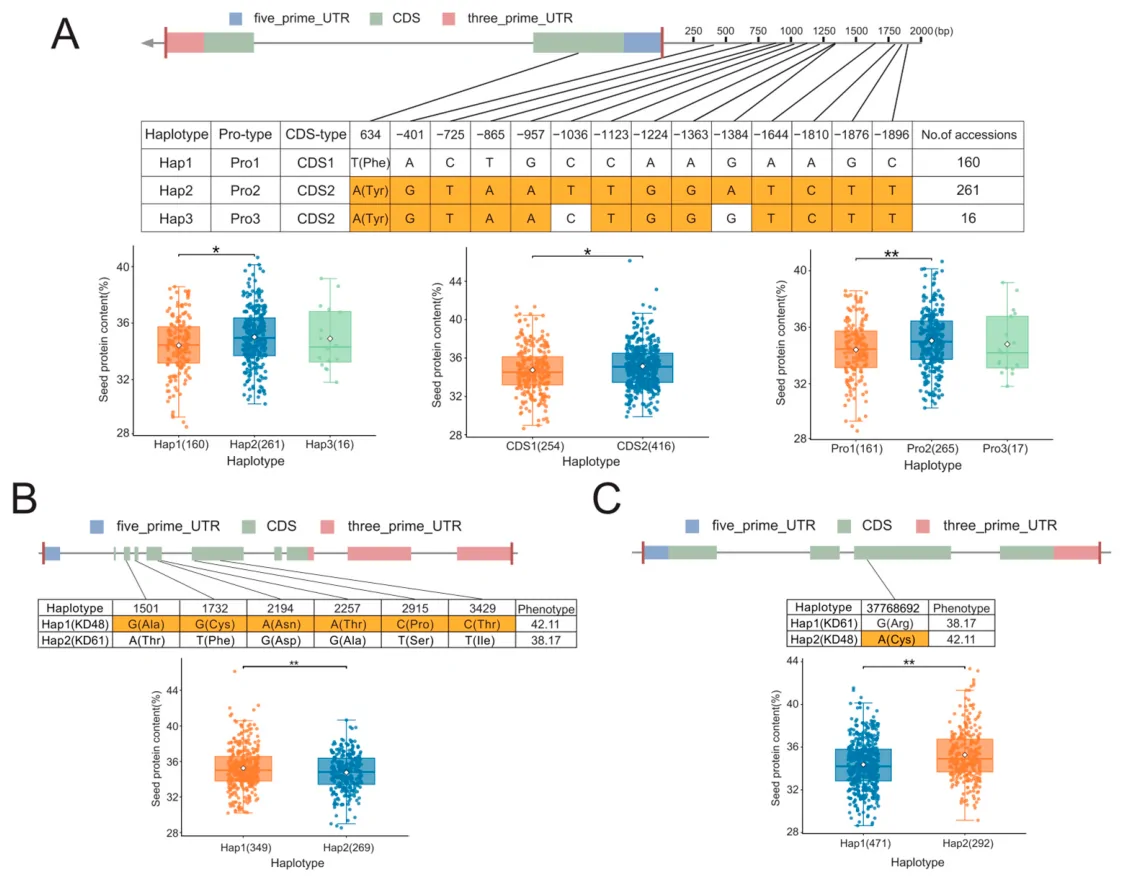
Haplotype phenotypic boxplots for three candidate genes. (**A**) *Glyma.08G246400* promoter and CDS haplotypes; (**B**) *Glyma.19G224000* CDS haplotypes; (**C**) *Glyma.09G154600* CDS haplotypes. Dots show individual accessions; box lines, medians; boxes, interquartile ranges; whiskers, values within 1.5 × the interquartile range; and white diamonds, means. Asterisks indicate statistical significance (* *p* < 0.05; ** *p* < 0.01).

Observed haplotype distributions are shown in Figure 6, and germplasm-type-adjusted associations are shown in Figure S8. For *Glyma.08G246400*, one CDS non-synonymous substitution and 13 promoter variants formed three integrated haplotypes. After adjustment for germplasm type, the integrated haplotype effect was significant (*n* = 437, adjusted *p* = 0.0012), with Hap2 showing a higher adjusted seed protein content than Hap1. *Glyma.09G154600* contained one missense variant defining Hap1 (G) and Hap2 (A); Hap2 had the higher adjusted mean (*n* = 763, adjusted *p* = 0.0018). *Glyma.19G224000* contained six CDS variants defining Hap1 (GGAACC) and Hap2 (ATGGTT); Hap1 had the higher adjusted mean (*n* = 618, adjusted *p* = 0.0288). Thus, all three haplotype associations remained significant after accounting for the broad differences among Soja, landrace, and improved-cultivar backgrounds.

### 2.6. Phylogenetic Relationships, Protein Domains, and Residue Conservation

Full-length protein alignments and neighbor-joining analyses of representative homologs from 15 Fabaceae species placed each *Glycine max* candidate protein with its *Glycine soja* homolog with 100% bootstrap support. In *Glyma.08G246400*, p.Phe109Tyr was located in the AFP/NINJA repression region; the KD48 Tyr residue occurred in 60.0% of the homologs, whereas the KD61/Wm82 Phe residue occurred in 6.7% (Figure S4). In *Glyma.09G154600*, p.Ala266Val mapped to the extracellular LRR region, and the KD48 Val and KD61/Wm82 Ala residues occurred in 66.7% and 26.7% of the homologs, respectively (Figure S5). For *Glyma.19G224000*, p.Ala18Thr, p.Cys62Phe, p.Asn156Asp, and p.Thr177Ala mapped to the IF2 N-terminal region, p.Pro199Ser to the EF-Tu-like GTP-binding domain, and p.Thr370Ile to EF-Tu domain 2. The KD61/Wm82 residues predominated at p.Ala18Thr (73.3%), p.Asn156Asp (86.7%), and p.Pro199Ser (80.0%), whereas the KD48 residues predominated at p.Cys62Phe (80.0%), p.Thr177Ala (93.3%), and p.Thr370Ile (60.0%) (Figure S6). These site-specific conservation patterns and domain locations provide evolutionary context for prioritizing the parental substitutions, but do not by themselves demonstrate causal effects on seed protein content.

### 2.7. Haplotype Frequencies and Geographic Distribution

Haplotype frequencies among germplasm types and geographic distributions are shown in Figure S7. The higher-protein-associated Hap2 of *Glyma.08G246400* occurred in 33.33% of Soja, 54.85% of landraces, and 67.02% of improved cultivars; carriers were recorded mainly in Heilongjiang, Shanxi, and Henan. For *Glyma.09G154600*, Hap2 occurred in 76.19%, 39.69%, and 31.95%, respectively, with many carriers sampled in the Huanghuai region. For *Glyma.19G224000*, Hap1 occurred in 96.88%, 49.85%, and 60.00%, respectively, and carriers were recorded across Northeast China, North China, and Huanghuai. These patterns describe the sampled accessions and do not demonstrate selection or regional adaptation.

## 3. Discussion

### 3.1. Genetic Characteristics of the Biparental RIL and Multi-Environment Trait Performance

Soybean seed protein is a highly heritable quantitative trait shaped by combined genetic, environmental, and G × E interaction effects [10]. The 174-line F_5_RIL population used in this study exhibited continuous, normal seed protein distribution across four year–location environments at three northern Chinese field locations, with wide phenotypic variation (31.24–46.65%) suitable for QTN mapping. ANOVA confirmed significant genetic and environmental effects on seed protein, consistent with previous multi-location soybean quality trials [10]. The broad-sense heritability of 0.89 indicated genetic variance dominated trait expression, validating the reliability of our phenotypic dataset for GWAS. However, the highly significant G × E interaction also demonstrated that single-environment phenotyping cannot identify universally stable high-protein lines, justifying our multi-location experimental design. Notably, mean seed protein content in 25NH (35.83%) was 3.93–5.86 percentage points lower than in the other environments, despite use of the same RIL panel and uniform planting density and fertilizer rates. Temperature, water availability, and nitrogen supply during seed filling may jointly affect soybean seed composition [25,26]. However, because stage-specific environmental and phenological data were unavailable and Nehe was evaluated in only one year, this difference should be interpreted as a 25NH-specific response rather than a persistent location effect.

Population structure analyses via Admixture and PCA showed the RIL panel contained only two ancestral genetic components derived from the two parents, with no distinct subpopulations. Incorporating PC1–PC3 as covariates effectively eliminated population stratification artifacts in association models [19]. Despite the advantages of biparental RILs in controlling background noise and tracking parental alleles, the limited population size (174 lines) has inherent drawbacks: reduced power to detect minor-effect QTNs and overestimation of locus phenotypic contribution (Beavis effect) [27]. Additionally, the panel only contains allelic variants segregating between the two parents and lacks rare elite alleles present in natural germplasm panels, a limitation addressed in later validation perspectives.

### 3.2. Advantages and Reliability of Multi-Model Combined GWAS

Most previous soybean seed protein GWAS rely on a single statistical model or limited field environments, leading to low reproducibility of identified loci across trials [10]. This study integrated 11 complementary multi-locus algorithms implemented through the mrMLM package, GAPIT3, IIIVmrMLM, and Fast3VmrMLM to jointly scan the soybean genome using model-specific significance criteria. A total of 54 QTNs were detected, among which 16 loci met the predefined repeat-detection criterion and were operationally defined as stable QTNs. Twelve were supported by multiple models within a single environment, whereas four were repeatedly detected across environments. Therefore, the stable QTN designation reflects cross-model and/or cross-environment reproducibility and should not be interpreted as evidence of environmental stability for all 16 loci [28]. Thirty-three QTNs overlapped with previously published seed protein QTLs archived in SoyBase, confirming the authenticity of our association signals [29,30]. Cross-model filtering reduced reliance on associations supported by only one statistical model and improved the robustness of QTN prioritization [31]. The 16 stable QTNs were located on chromosomes 4, 8, 9, 12, 18, 19, and 20, identifying priority genomic regions for fine mapping and gene cloning; 21 loci without prior documentation represent novel protein-associated QTNs that expand existing soybean genomic resources for quality improvement.

### 3.3. Candidate Genes and Putative Functional Context

We constructed a multi-evidence screening pipeline integrating LD boundaries, seed expression, parental non-synonymous variants, and haplotype-association evidence to avoid overinterpreting isolated GWAS signals, a widely accepted strategy for soybean candidate gene identification [32]. The three candidate genes have putative annotations related to transcriptional repression, signal transduction, and translation, providing a proposed functional framework for seed protein variation [33]. These interpretations remain correlative, and causal roles require transgenic or gene-editing validation.

The comparative analysis provided independent evolutionary context for these candidates. Five KD48 residues were the predominant states among the sampled legume homologs, whereas the KD61/Wm82 residues predominated at the other three sites in *Glyma.19G224000*. This mixed pattern indicates site-specific evolutionary constraint rather than uniform conservation of either parental protein. Because all eight substitutions lie within annotated protein regions, they are suitable targets for functional testing; however, phylogenetic placement, residue conservation, and domain location alone do not establish causality.

#### 3.3.1. Glyma.08G246400: Candidate AFP3/NINJA-like Transcriptional Repressor

This gene encodes an AFP3/NINJA transcriptional repressor in the ABA signaling cascade. Homologous AFP proteins in *Arabidopsis* interact with ABI5 to suppress ABA-mediated seed maturation gene expression [34]. Vittozzi et al. (2024) further showed that AFP proteins recruit TOPLESS co-repressors to constrain ABA-responsive transcription, while Ju et al. (2019) reported JAZ–ABI5 interactions that repress ABA-dependent transcriptional programs [34,35]. These observations support a role for transcriptional repression modules in coordinating seed maturation-related gene expression. Our data showed the gene is highly expressed in developing soybean seeds and carries non-synonymous substitutions between the two parents. After adjustment for germplasm type, Hap2 remained associated with higher seed protein content. Its frequency increased from Soja to improved cultivars, but this descriptive pattern does not establish selection or causality.

#### 3.3.2. Glyma.09G154600: Candidate LRR Receptor-like Protein Kinase

LRR-RLK proteins act as plasma membrane receptors to transduce extracellular nutrient signals via intracellular phosphorylation cascades [36]. Phosphoproteomic assays under carbon/nitrogen starvation confirm LRR-RLKs mediate metabolic reprogramming in plant cells [36]. These established roles of LRR-RLKs in signal perception and nutrient-responsive metabolic reprogramming provide functional context for a potential association of *Glyma.09G154600* with seed composition variation. After adjustment for germplasm type, Hap2 remained associated with higher seed protein content. It was less frequent in landraces and improved cultivars than in Soja, but this pattern does not demonstrate allele loss during domestication. Hap2 therefore warrants evaluation in cultivated genetic backgrounds.

#### 3.3.3. Glyma.19G224000: Candidate Translation Initiation Factor IF-2

IF-2/eIF5B mediates ribosomal subunit assembly to control the global rate of cellular protein synthesis [37]. This established role in translation initiation provides functional context for the association of *Glyma.19G224000* with seed protein content but does not establish a causal role in storage-protein synthesis. After adjustment for germplasm type, Hap1 remained associated with higher seed protein content; its occurrence in all three germplasm categories supports further evaluation but does not demonstrate regional adaptation. Collectively, their annotations suggest a proposed framework linking transcriptional repression, nutrient-signal perception, and translation; this hypothesis requires functional validation.

### 3.4. Haplotype Frequency Patterns and Breeding Implications

Haplotype frequencies differed among Soja, landraces, and improved cultivars, but these differences do not establish directional selection or allele loss [27]. The haplotype–protein associations remained significant after including germplasm type as a covariate (Figure S8). *Glyma.08G246400* Hap2 was more frequent in improved cultivars, whereas *Glyma.09G154600* Hap2 was more frequent in Soja. Their geographic distributions identify materials for further multi-environment evaluation but do not demonstrate regional adaptation. A recent study of Northeast China soybean germplasm likewise used haplotype analysis to prioritize seed-composition alleles [38]. The non-synonymous SNPs in the three genes are potential targets for KASP marker development and validation [39].

### 3.5. Limitations and Future Research Directions

This study systematically characterizes seed protein QTNs and candidate genes via multi-environment phenotyping and multi-model GWAS, yet several limitations remain: (1) RIL population constraints: small panel size (174 lines) limits minor QTN detection and introduces Beavis effect; the population lacks natural rare alleles. Future work will deploy large-scale NAM or natural germplasm panels to validate locus effects; (2) absence of gene functional validation: current evidence is correlative (GWAS, expression, haplotypes). Follow-up qRT-PCR, near-isogenic line phenotyping, CRISPR knockout, and overexpression transformation will verify causal gene functions [40]; (3) limited environmental coverage: only four year–location environments at three northern Chinese locations were evaluated. Additional multi-year trials in Huanghuai and southern soybean regions are required to evaluate QTN-wide adaptability; (4) missing gene epistasis analysis: this study only analyzed single-haplotype independent effects. Haplotype pyramiding lines will be constructed to test multi-gene additive effects; (5) evolutionary inference: haplotype-frequency and geographic patterns were descriptive because formal selection scans and environmental association tests were not performed.

Two primary research directions are proposed: (1) mechanistic validation through gene editing, interaction assays, and transcriptomics; and (2) breeding evaluation through validation of gene-specific KASP markers, testing of protein-associated haplotype combinations in early-maturing lines, and evaluation of *Glyma.09G154600* Hap2 in cultivated backgrounds.

## 4. Materials and Methods

### 4.1. Plant Materials and Population Construction

Two early-maturing spring soybean cultivars (Maturity Group 00, MG 00) with contrasting seed protein phenotypes, Kedou 48 (KD48, 44.34% seed protein, high-protein parent) and Kedou 61 (KD61, 38.76% seed protein, low-protein parent), were selected to build the segregating RIL population. Both cultivars were bred and preserved at the Keshan Branch, Heilongjiang Academy of Agricultural Sciences, and officially released for high-latitude northern Heilongjiang planting regions, which require ultra-early maturity to complete seed filling before frost. KD48 was derived from the cross Kejiao 99-578 × Dongnong 50; KD61 was developed from Keshan 1 × Suinong 26. The derived F_5_RIL population segregates for seed protein content while maintaining the early-maturing genetic background adapted to high-latitude growing environments.

The single seed descent (SSD) method was applied to generate the F_5_ RIL population [41]. The KD48 × KD61 cross was completed in Keshan in 2021 to produce F_1_ seeds. Hainan winter nurseries were used to accelerate generational advancement: F_1_ (2021 winter), F_3_ (2022 winter), and F_5_ (2023 winter) generations were planted sequentially, while F_2_ and F_4_ lines were grown in Keshan during the 2022 and 2023 growing seasons, respectively. Individual F_5_ plants were harvested in Hainan winter 2023 to form a population of 174 homozygous lines (homozygosity > 95%), which was exclusively used for multi-environment phenotyping and GWAS.

The ZH13.v2-referenced VCF used for haplotype analysis was obtained with permission through a previous collaborative project involving our research group. The 2898 accessions correspond to the SoyOmics Variome panel [42], but the complete VCF used here was supplied through the collaboration rather than downloaded from SoyOmics. Seed protein-content values were the best linear unbiased prediction (BLUP) values provided by the SoyOmics Phenome resource for multi-year or multi-location records, and germplasm-type metadata were obtained from the SoyOmics accession records. Because only accessions with homozygous target-gene haplotypes and complete protein-content and germplasm-type records were retained, the effective sample size differed among genes. These data were used for haplotype–phenotype, germplasm-composition, and geographic-distribution analyses (Section 2.5 and Section 2.7).

### 4.2. Field Experimental Design and Seed Protein Phenotyping

To evaluate environmental and genotype-by-environment (G × E) effects on seed protein content and assess the repeatability of QTN detection, the 174 RILs were evaluated in four year–location environments: Keshan in 2024 (24KS), Keshan in 2025 (25KS), Harbin in 2025 (25HRB), and Nehe in 2025 (25NH). Within each environment, a randomized complete block design with three biological replicates was used. Each replicate consisted of a single 5 m row with 0.65 m row spacing. A planting density of 30 × 10^4^ plants ha^−1^ and identical N/P_2_O_5_/K_2_O fertilizer rates were applied across environments. Site coordinates, sowing dates, planting density, and fertilizer application rates are provided in Table S6 [10].

At physiological maturity, 10 uniform, lodging- and disease-free plants were harvested from the central portion of each plot to minimize border effects. Seeds were air-dried until their moisture content stabilized at approximately 13%. Seed protein content was determined using a FOSS 1241 near-infrared grain analyzer (FOSS Tecator, Höganäs, Sweden) calibrated with certified soybean reference samples. Each plot sample was measured three times, and the technical measurements were averaged to obtain a plot-level value. For each RIL within each environment, the arithmetic mean of the three biological-replicate plot values was used as the environment-specific phenotype for GWAS [7].

### 4.3. Statistical Analysis of Phenotypic Data

Phenotypic statistical analyses were performed using SAS 9.2 (SAS Institute Inc., Cary, NC, USA) and R v4.2.1 (R Foundation for Statistical Computing, Vienna, Austria). The Shapiro–Wilk test was used to verify the normal distribution of seed protein content within each environment, a prerequisite for GWAS. Descriptive statistics (mean, standard deviation, coefficient of variation, maximum, minimum, range, skewness, kurtosis) were calculated via the R psych package; violin plots illustrating trait distribution were generated with ggplot2 [43].

A mixed linear model was fitted using the SAS PROC MIXED procedure to partition phenotypic variance. Environment was set as a fixed factor, while genotype and G × E interaction were designated random variables. The formula for broad-sense heritability (h^2^) is as follows:

$$h^{2}=\frac{\sigma_{G}^{2}}{\sigma_{G}^{2}+\frac{\sigma_{\mathrm{GE}}^{2}}{E}+\frac{\sigma_{e}^{2}}{\mathrm{ER}}}$$

where $\sigma_{G}^{2}$ = genotypic variance, $\sigma_{\mathrm{GE}}^{2}$ = G × E interaction variance, $\sigma_{e}^{2}$ = residual error variance, E = number of test environments, R = number of field replicates. Variance components extracted from the mixed model were used to calculate heritability and perform ANOVA to test the significance of genetic and environmental effects [10].

### 4.4. Genotyping and Population Structure Analysis

Young leaf tissues were sampled from each RIL line, snap-frozen in liquid nitrogen, and ground for genomic DNA extraction via the standard CTAB protocol. High-density genotyping was completed by Beidahuang Kenfeng Seed Co., Ltd. (Harbin, China) using the SoySNP180K array. The physical positions supplied for the array markers, and hence the coordinates assigned to the QTNs identified by GWAS, were referenced to the *Glycine max* Wm82.a2.v1 assembly.

Of 180,961 assayed probe sets, the genotyping provider delivered genotype calls for 169,388 markers across the 174 RILs. This 169,388-marker dataset was subjected to downstream QC using PLINK 2.0. Stringent quality control (QC) filters were applied to raw SNP markers: markers with zero homozygous AA/BB genotype frequency, minor allele frequency (MAF) < 0.05, or missing data rate > 20% were discarded. A final set of 25,663 high-quality SNPs was retained for population stratification correction and GWAS.

Principal component analysis (PCA) was implemented in PLINK v1.9 using filtered SNP data [44]. The first three principal components (PC1–PC3) were extracted as covariates to mitigate population stratification bias in association models. ADMIXTURE v1.3.1 was used to resolve ancestral genetic components, and CV errors were compared for K = 1–10 (Figure S1). Although the absolute minimum CV error occurred at K = 10, the largest decrease was observed between K = 1 and K = 2. Considering the known biparental origin of the RIL population, K = 2 was selected as the most parsimonious model for visualizing the two parental ancestry components [45]. An ancestry bias index was defined as Q_1_ − Q_2_ (Q_1_ = KD48 ancestry proportion, Q_2_ = KD61 ancestry proportion). Lines with bias index > 0.3 were classified as KD48-like; values < −0.3 were KD61-like; lines ranging from −0.3 to 0.3 were categorized as balanced ancestry. This classification was only used for visualization and not incorporated into GWAS statistical models.

### 4.5. Multi-Locus GWAS Implementation

Population structure and relatedness were handled according to the statistical framework of each GWAS model. PC1–PC3 were included as covariates in all 11 models. For GAPIT, the genome-wide kinship matrix used by MLMM was calculated internally using the VanRaden algorithm specified by kinship.algorithm = “VanRaden”; FarmCPU iteratively derived a model-specific kinship matrix from selected pseudo-QTNs, whereas BLINK did not fit a kinship matrix. For the six mrMLM-package methods and the 3VmrMLM (implemented in IIIVmrMLM) and Fast3VmrMLM analyses, fileKin = NULL was used, so relatedness was handled, where required, by the internal procedures of the corresponding software [19,20,21,22,23,46,47]. For the three GAPIT3 models, a Bonferroni-corrected *p*-value threshold was used for genome-wide significant QTNs (*p* < 0.05/25663 = 1.95 × 10^−6^); a relaxed *p*-value threshold (*p* < 1.0 × 10^−5^) was used only for suggestive associations entering the repeat-detection screen. A QTN meeting the corresponding model-specific screening criterion and detected by at least two GWAS models within the same environment and/or in at least two environments was operationally defined as a stable QTN for candidate-gene mining [46]. QTNs meeting only the cross-model criterion were not interpreted as environment-stable; cross-environment repeatability was assigned only to QTNs detected in at least two environments [28].

Three categories of multi-locus algorithms were used. The mrMLM series (mrMLM v4.0.2 R package) comprised mrMLM, FASTmrMLM, FASTmrEMMA, pLARmEB, pKWmEB, and ISIS EM-BLASSO, with PopStrType = “PCA”; QTNs with a final LOD score ≥ 3.0 were retained, and a 100-kb scanning window was used for mrMLM and FASTmrMLM [19,47]. The GAPIT3 analyses comprised BLINK, FarmCPU, and MLMM, with PCA.total = 3 and additive SNP effects; significance was assessed from the *p*-values reported by GAPIT3, and the corresponding Table S2 statistics are −log_10_(*p*), not LOD scores [20,21,46]. The 3V analyses comprised 3VmrMLM, implemented in IIIVmrMLM with a 20-kb search radius, and Fast3VmrMLM (20-kb window and eight parallel threads); the software-implemented two-stage procedure used the Bonferroni-corrected *p*-value threshold for significant associations and a final LOD score ≥ 3.0 for suggested associations [22,23].

Physical coordinates of all detected QTNs were aligned with published seed protein QTLs archived in SoyBase. MapChart 2.32 was used to generate chromosomal distribution maps for all detected QTNs [48]. Accordingly, QTN–QTL comparisons and chromosome-map visualization were performed in the Wm82.a2.v1 coordinate system, and the QTN positions reported in the manuscript and Supplementary Tables remain Wm82.a2.v1 coordinates.

### 4.6. Stepwise Candidate Gene Prediction

Genome-wide LD decay was calculated using PopLDdecay v3.43 [49] with a maximum pairwise distance of 5000 kb; bin1 and bin2 were set to 1000 and 10,000 bp, respectively, and a pair-count-weighted LOESS curve was fitted. The first post-peak distance at which fitted mean r^2^ declined to 50% of its fitted maximum was 1674.2 kb (Figure 5A). Because this long-range LD in the biparental RIL population [41] would yield a megabase-scale gene search, it was reported descriptively but was not used to define the candidate interval. Candidate genes were instead prioritized within ±100 kb of each stable QTN (200-kb total span), consistent with soybean GWAS practice [24]. A 100-kb total span (±50 kb) was considered too restrictive because the 50–100 kb segment retained high fitted mean r^2^ and still showed substantial LD decline (Figure 5B,C). Thus, the 200-kb span was treated as a stringent operational prioritization interval rather than the genome-wide LD decay distance. Candidate genes within the QTN intervals were assigned using Wm82.a2.v1 gene models. Gene counts were based on unique IDs across overlapping QTN-centered intervals. Sequential filtering steps were performed: (1) retain all genes within ±100 kb of each stable QTN (200-kb total span); (2) retain genes whose FPKM was ≥ 5 in at least one of the five developing-seed stages (seed-1 to seed-5) in the SoyOmics tissue-expression dataset [42]; stage-specific FPKM values were evaluated separately rather than averaged; (3) screen genes carrying non-synonymous missense mutations in the independently generated KD48 and KD61 parental resequencing data, removing synonymous and intronic variants; these parental data were separate from the collaborative 2898-accession panel corresponding to the SoyOmics Variome resource; (4) perform haplotype–phenotype association tests with germplasm type included as a covariate; genes showing a significant adjusted haplotype effect (*p* < 0.05) were designated final candidate genes. Before analysis of the ZH13.v2-based resequencing panel, the Wm82.a2.v1 candidate-gene IDs were matched to their corresponding ZH13.v2 gene models. No genome-wide conversion of QTN coordinates was performed; cross-assembly correspondence was applied only to the candidate genes selected after the Wm82.a2.v1-based GWAS. The paired Wm82.a2.v1 and ZH13.v2 identifiers are provided in Table S3.

Functional annotation and Gene Ontology (GO) term assignment for the candidate genes were performed using eggNOG-mapper against the eggNOG orthology database [50]; the assigned GO terms were used to summarize their putative functions, and no statistical GO enrichment analysis was performed. For heatmap visualization, the 19 genes with zero FPKM variance across tissues were omitted, and FPKM values for the remaining 160 genes were transformed to row-wise Z scores across tissues. Genes were hierarchically clustered using Euclidean distance and complete linkage, whereas tissues were displayed in a predefined order without clustering (Figure S3) [32].

### 4.7. Haplotype Characterization of Candidate Genes

Haplotype analysis was conducted locally and did not use the SoyOmics HapSnap module. Target-gene variants were extracted from the collaborative ZH13.v2-referenced VCF of the 2898-accession panel; this panel was not the source of the KD48–KD61 parental variants. After the three final candidate genes had been identified in Wm82.a2.v1 coordinates, their gene IDs were matched to the corresponding ZH13.v2 gene models. The Wm82.a2.v1 and ZH13.v2 target-gene sequences were aligned in SnapGene to verify sequence correspondence and the ZH13.v2 intervals used for extraction. Variants within the 2000-bp upstream promoter and full-length coding sequence (CDS) were extracted with VCFtools under Linux. Only homozygous variant calls were retained for haplotype assignment; accessions with heterozygous or missing target-site calls, or without matched seed protein BLUP or germplasm-type information, were excluded. The resulting effective sample sizes were 443, 670, and 437 for the *Glyma.08G246400* promoter, CDS, and integrated haplotypes, respectively; 763 for *Glyma.09G154600*; and 618 for *Glyma.19G224000*. Haplotype construction and visualization were performed locally using tabulated data and R. To account for broad genetic-background differences, seed protein BLUP was modeled as a function of haplotype and germplasm type (Soja, landrace, or improved cultivar). Haplotype effects were evaluated by Type II tests with HC3 heteroskedasticity-robust covariance; adjusted marginal means were equally weighted across germplasm types, and Tukey-adjusted pairwise comparisons were used where applicable. Haplotype-by-type interaction and within-type analyses were used as sensitivity checks when estimable. Haplotype frequencies and geographic distributions were summarized separately in R.

### 4.8. Phylogenetic, Domain, and Residue Conservation Analyses

To assess amino-acid conservation at parental missense variant sites, representative homologs of *Glyma.08G246400*, *Glyma.09G154600*, and *Glyma.19G224000* were retrieved from 15 Fabaceae reference proteomes available through LegumeInfo/SoyBase. Gene and protein identifiers, annotation versions, and sequence sources for all homologs used in these analyses are listed in Table S7. One homolog per species was selected using Legume.fam3 family assignments, full-length protein identity, query coverage, and length similarity; when a family assignment was unavailable, complete-proteome candidates were prescreened by amino-acid 3-mer similarity and the best full-length match was retained. Protein sequences were aligned with FAMSA v0.7.0, and neighbor-joining trees were reconstructed from full-length protein p-distances with 500 bootstrap replicates. Missense variants identified from the independently generated KD61 and KD48 parental resequencing data were mapped to Wm82.a2 protein positions and the corresponding alignment columns. At each site, the proportions of homologs carrying the KD61/Wm82 residue, the KD48 residue, or another amino acid were calculated. Protein regions were assigned from Pfam annotations associated with the Wm82.a2 proteins and sequence landmarks, and each missense substitution was mapped to the corresponding annotated region.

## 5. Conclusions

Using a biparental early-maturing soybean RIL population phenotyped across four field environments and 11 complementary multi-locus GWAS algorithms, we identified 16 stable seed protein QTNs under the predefined cross-model and/or cross-environment repeat-detection criterion, including 12 supported by multiple models within a single environment and four detected across at least two environments. These QTNs were located on chromosomes 4, 8, 9, 12, 18, 19, and 20. Within the broader six-step workflow summarized in Table 2, four candidate-gene screening steps—LD-interval gene retrieval, developing-seed expression filtering, parental non-synonymous variant screening, and haplotype–phenotype association testing—prioritized three candidate genes (*Glyma.08G246400*, *Glyma.09G154600*, and *Glyma.19G224000*) with putative annotations related to transcriptional repression, signal transduction, and translation, respectively.

Haplotype analyses of eligible subsets drawn from the 2898-accession collaborative panel detected associations for all three genes after adjustment for germplasm type and revealed divergent haplotype-frequency and geographic patterns. This study provides stable genomic loci, candidate genes, and protein-associated haplotypes for further mechanistic and breeding evaluation in high-protein early-maturing soybean.

## Figures and Tables

**Table 1:** ANOVA table and broad-sense heritability estimation for seed protein content.

Source	DF	SS	MS	F	Pr > F	Variance Component
Environment	3	10,321.18	3440.39	990.67	<0.0001	6.58
Block (Environment)	8	0.0061	0.0007	0.49	0.86	—
Genotype	173	5834.07	33.72	9.71	<0.0001	2.53
Environment × Genotype	513	1781.53	3.47	2262.69	<0.0001	1.15
Error	1372	2.11	0.0015			0.0015
Total	2069	17,938.91				
h^2^						0.89

ANOVA results (Table 1) revealed highly significant (*p* < 0.0001) genotype, environment, and G × E interaction effects on seed protein content. Based on decomposed variance components, the broad-sense heritability of seed protein content reached 0.89, confirming that genetic factors dominated phenotypic variation and validating the reliability of multi-environment phenotypic data.

**Table 2:** Stepwise hierarchical screening pipeline for candidate genes.

Step	Processing	Screening Output
Step 1	Multi-locus GWAS mapping	54 total QTNs
Step 2	Repeat-detection filtering across models and/or environments	16 stable QTNs
Step 3	Strict candidate-gene prioritization within ±100 kb of each stable QTN (200-kb total span)	179 candidate genes
Step 4	Filtering for high seed expression	40 genes with FPKM ≥ 5 in at least one of five developing-seed stages
Step 5	Screening for parental non-synonymous CDS mutations	7 unique mutated genes
Step 6	Assessment of haplotype–phenotype associations	3 final candidate genes

## Data Availability

Restrictions apply to the availability of the ZH13.v2-referenced VCF for the 2898-accession panel. The VCF was provided for research use through a previous collaborative project and is not authorized for public redistribution without permission from the data provider. Requests may be directed to the corresponding authors and require permission from the data provider. Public descriptions of the panel, accession records, and phenotype records are available in SoyOmics (https://ngdc.cncb.ac.cn/soyomics/documentation (accessed on 13 October 2026); phenome module: https://ngdc.cncb.ac.cn/soyomics/phenome/ (accessed on 13 October 2026)) [42]. The remaining original contributions are included in the article and Supplementary Materials.

## Supplementary Materials

The following supporting information can be downloaded at https://www.mdpi.com/article/10.3390/plants15213298/s1, Figure S1: Cross-validation error values of ADMIXTURE analysis with different numbers of assumed ancestral populations (K = 1–10); Figure S2: Quantile–quantile (QQ) plots comparing observed and expected −log_10_(*p*) values for seed protein content; Figure S3: Tissue expression heatmap of the 160 candidate genes with nonzero FPKM variance across tissues; Figure S4: Phylogenetic relationships, protein architecture, and cross-species residue conservation of *Glyma.08G246400* and the parental p.Phe109Tyr (F109Y) substitution among 15 Fabaceae species; Figure S5: Phylogenetic relationships, protein architecture, and cross-species residue conservation of *Glyma.09G154600* and the parental p.Ala266Val (A266V) substitution among 15 Fabaceae species; Figure S6: Phylogenetic relationships, protein architecture, and cross-species residue conservation of *Glyma.19G224000* and six parental missense substitutions among 15 Fabaceae species; Figure S7: Haplotype frequency and geographic distribution; Figure S8: Germplasm-type-adjusted haplotype associations with seed protein content and haplotype composition for three candidate genes; Table S1: Descriptive statistics of seed protein content across test environments; Table S2: QTNs associated with soybean protein content were identified; Table S3: Functional annotation of 179 candidate genes and corresponding ZH13.v2 gene IDs; Table S4: Details of seven unique genes with nonsynonymous mutations; Table S5: GO functional annotation of the candidate genes *Glyma.08G246400*, *Glyma.19G224000*, and *Glyma.09G154600*; Table S6: Field cultivation conditions for the RIL population across four test environments; Table S7: Gene and protein identifiers of homologs used in the phylogenetic and amino-acid conservation analyses. The seven source studies cited in Table S2 are included in the main reference list [51,52,53,54,55,56,57] in the same order.
