# Supplementary material for: Multi-Locus Genome-Wide Association Study Identifies Stable Quantitative Trait Nucleotides and Candidate Genes for Seed Protein Content in an Early-Maturing Soybean Recombinant Inbred Line Population
Source: Plants (Basel). 2026 Oct 26;15(21):3298. doi: 10.3390/plants15213298 (PMC13766170; doi:10.3390/plants15213298)
Supplement: Supplementary file 1 [file plants-15-03298-s001.zip › Supplementary_Figures.pdf]

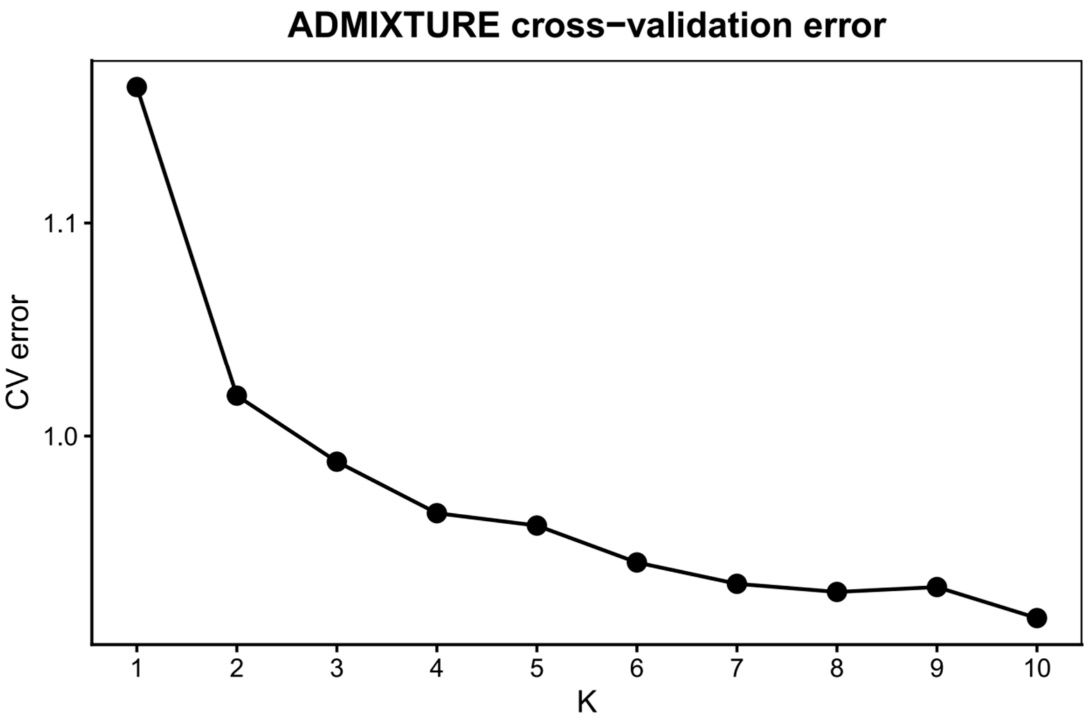

Figure S1. Cross-validation error values of ADMIXTURE analysis with different numbers of assumed ancestral populations (K = 1–10).

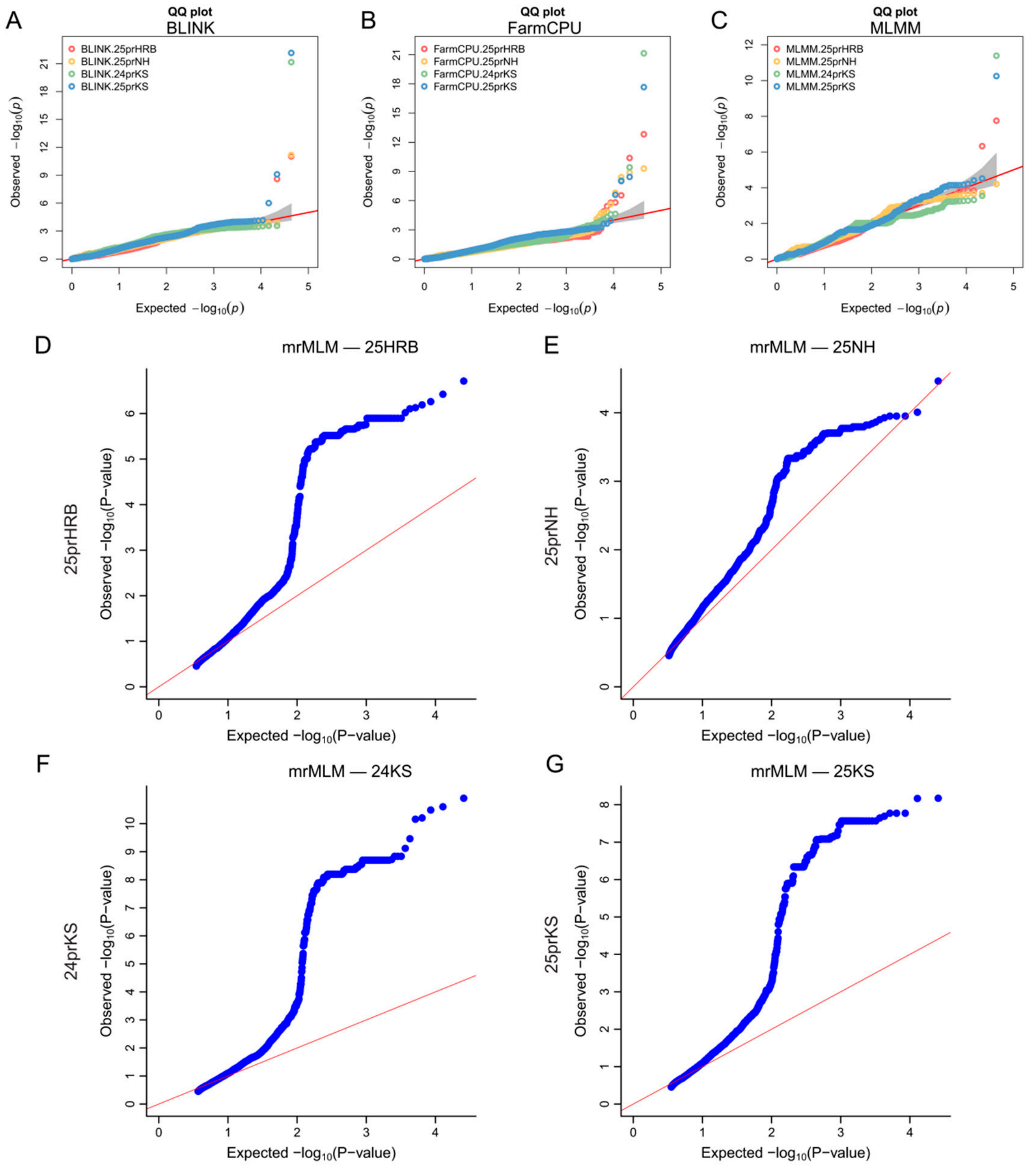

Figure S2. Quantile–quantile (QQ) plots comparing observed and expected $-\log_{10}(P)$ values for seed protein content. Panels (A–C) show results from BLINK, FarmCPU, and MLMM, respectively. In these panels, red, orange, green, and blue points represent 25HRB, 25NH, 24KS, and 25KS, respectively. Panels (D–G) show results from the mrMLM algorithm for 25HRB, 25NH, 24KS, and 25KS, respectively; blue points represent the observed values in each panel. The red diagonal lines indicate equality between observed and expected values. Environment abbreviations: 25HRB, Harbin in 2025; 25NH, Nehe in 2025; 24KS, Keshan in 2024; and 25KS, Keshan in 2025.

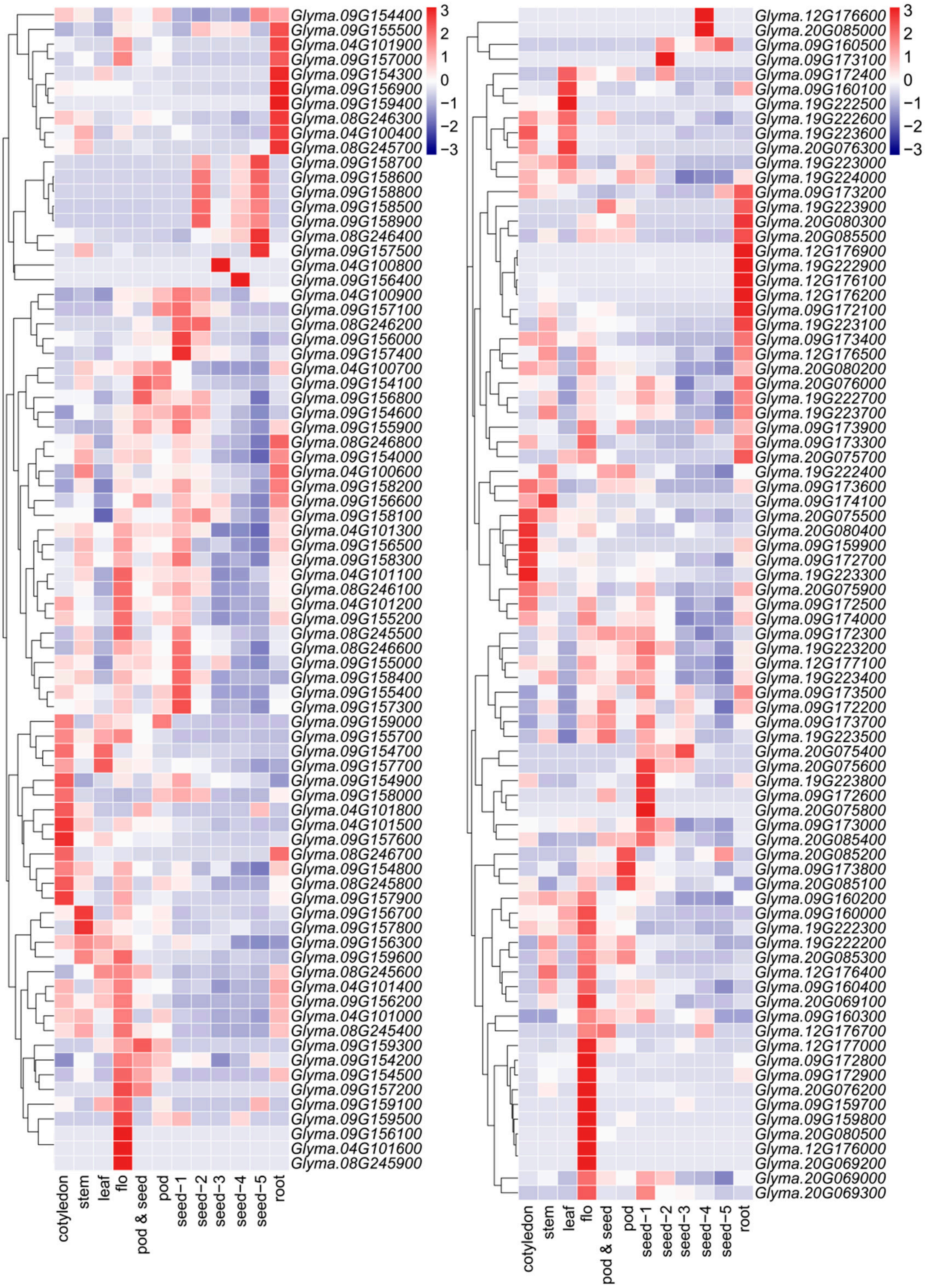

Figure S3. Tissue expression heatmap of the 160 candidate genes with nonzero FPKM variance across tissues. FPKM values were standardized to row-wise Z scores across tissues. Genes were hierarchically clustered using Euclidean distance and complete linkage, whereas tissues were displayed in a predefined order without clustering.

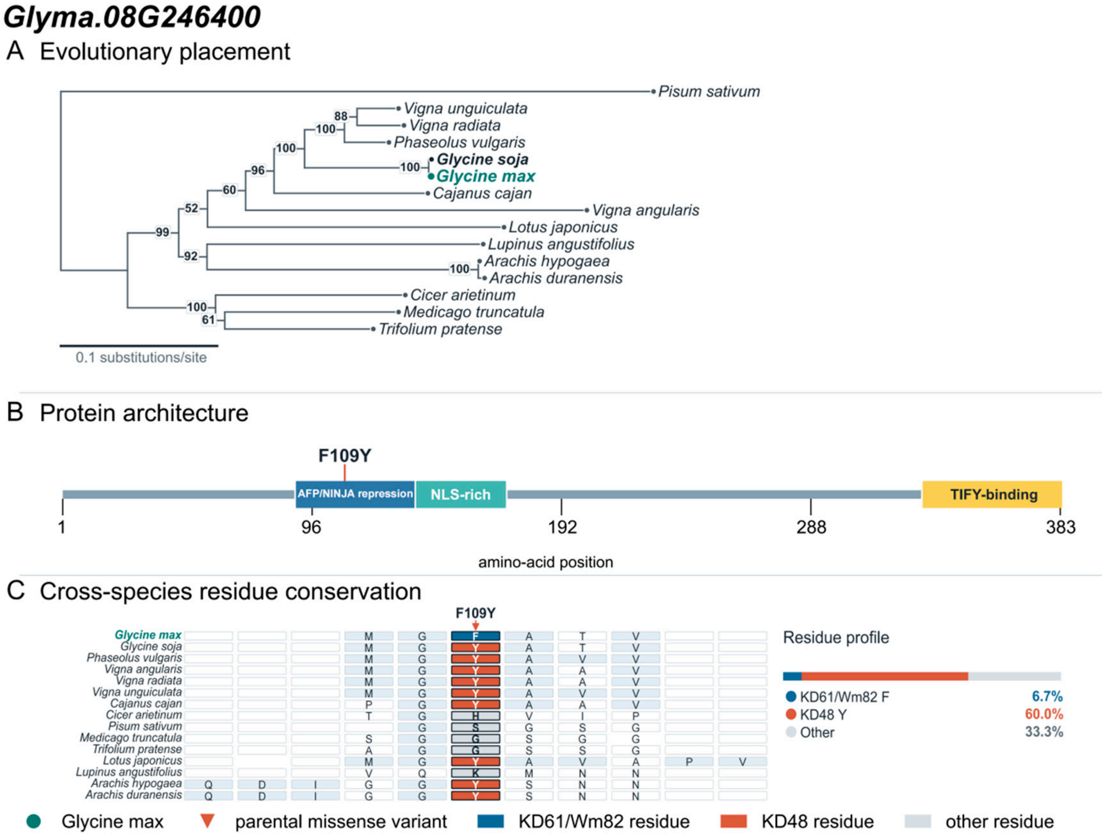

Figure S4. Phylogenetic relationships, protein architecture, and cross-species residue conservation of *Glyma.08G246400* and the parental p.Phe109Tyr (F109Y) substitution among 15 Fabaceae species.

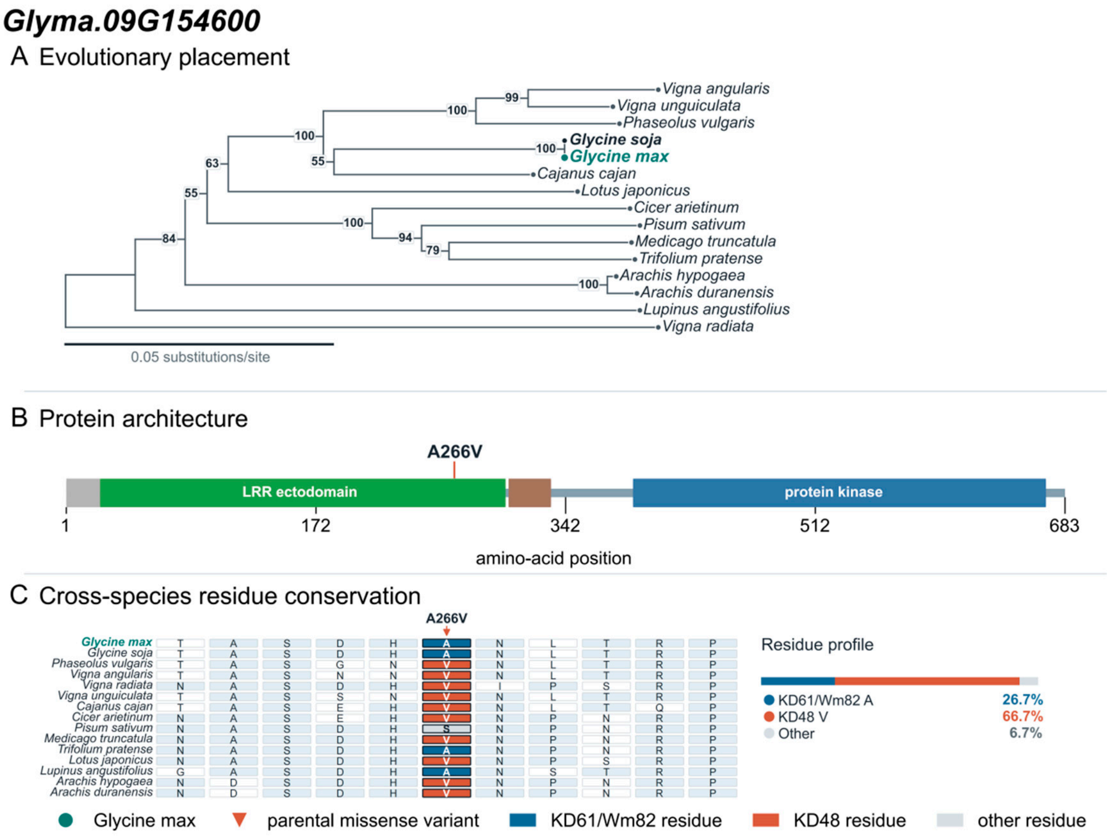

Figure S5. Phylogenetic relationships, protein architecture, and cross-species residue conservation of *Glyma.09G154600* and the parental p.Ala266Val (A266V) substitution among 15 Fabaceae species.

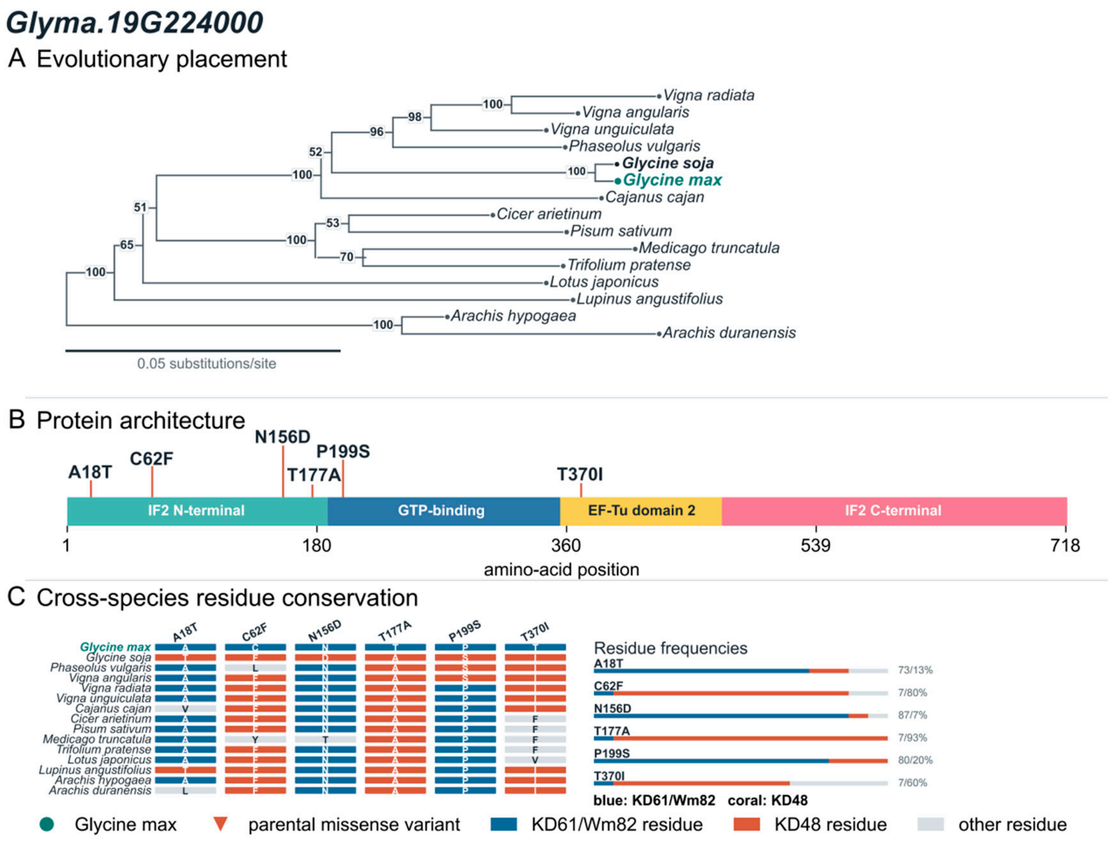

Figure S6. Phylogenetic relationships, protein architecture, and cross-species residue conservation of *Glyma.19G224000* and six parental missense substitutions (p.Ala18Thr, p.Cys62Phe, p.Asn156Asp, p.Thr177Ala, p.Pro199Ser, and p.Thr370Ile) among 15 Fabaceae species.

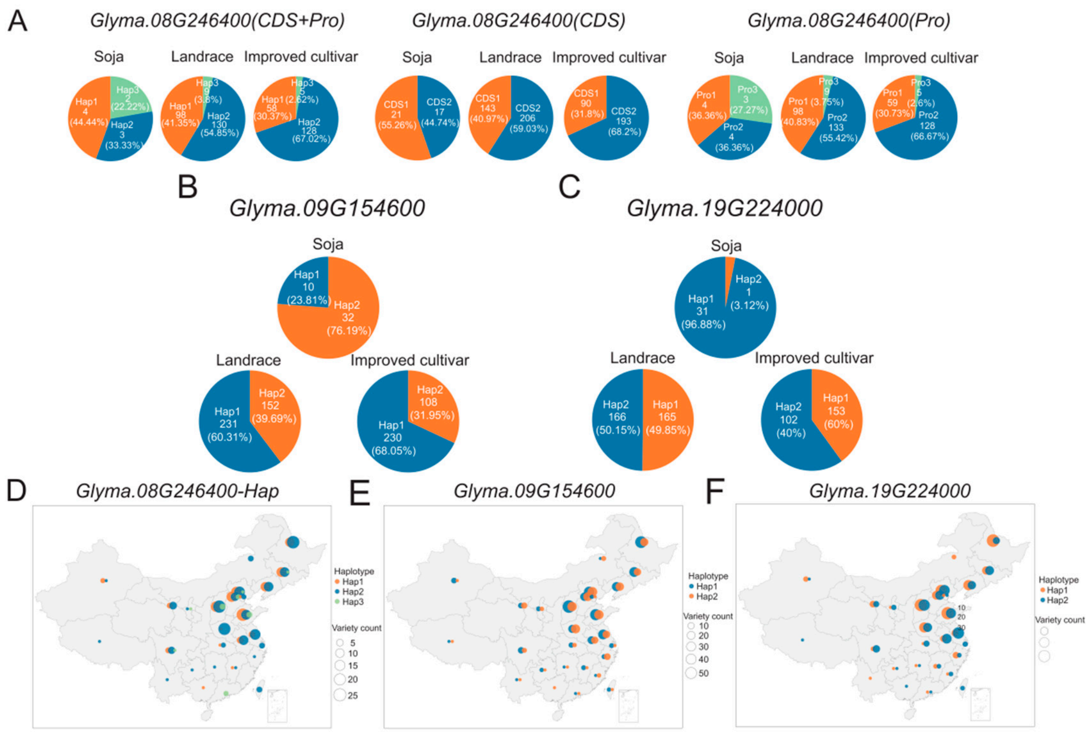

Figure S7. Haplotype frequency and geographic distribution. (A－C) Pie charts of haplotype proportions in wild soybeans, landraces, and elite cultivars for each gene; (D－F) National geographic bubble maps showing germplasm distribution of each major haplotype.

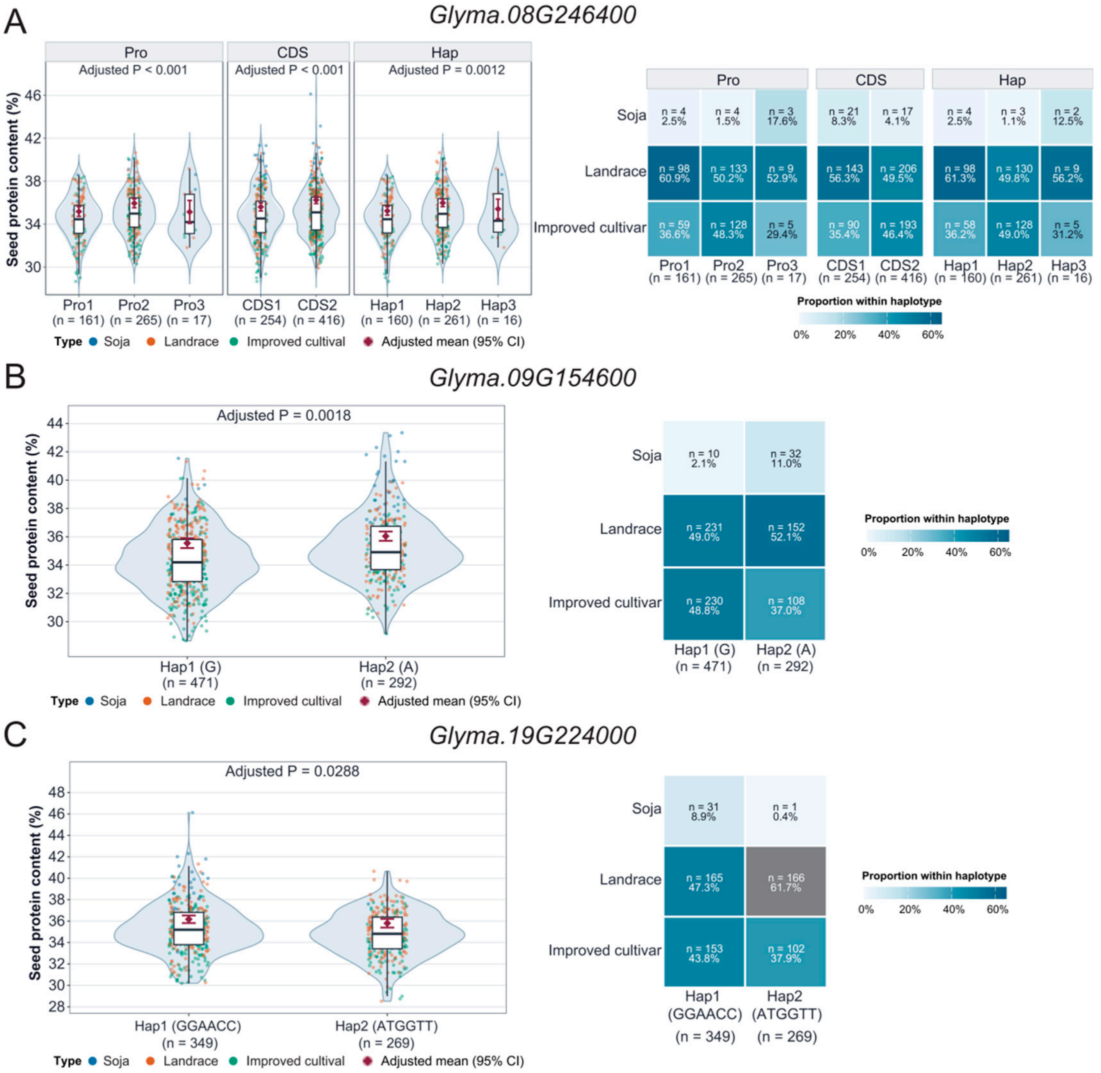

Figure S8. Germplasm-type-adjusted haplotype associations with seed protein content and haplotype composition for three candidate genes. (A) Promoter (Pro), coding-sequence (CDS), and integrated promoter–CDS (Hap) haplotypes of *Glyma.08G246400*; (B) CDS haplotypes of *Glyma.09G154600*; and (C) CDS haplotypes of *Glyma.19G224000*. Violin and box plots show the distributions of the seed protein-content BLUP values obtained from SoyOmics. Point colors indicate germplasm type—Soja, landrace, or improved cultivar—and diamonds with error bars represent germplasm-type-adjusted marginal means and their 95% confidence intervals. Heatmaps show the germplasm composition of each haplotype; values indicate the number and proportion of accessions within each haplotype. Adjusted P values were obtained from Type II tests of the haplotype effect in linear models including germplasm type, with HC3 heteroskedasticity-robust covariance. Only accessions with homozygous target-gene haplotypes and complete seed protein-content and germplasm-type information were included.
